# Study of Nonculture Epidermal Suspension in the Treatment of Stable Vitiligo

**DOI:** 10.1055/s-0045-1806766

**Published:** 2026-01-29

**Authors:** Prashant Baranwal

**Affiliations:** 1 Department of Plastic Surgery, Banaras Plastic Surgery Hospital, Varanasi, Uttar Pradesh, India

**Keywords:** stable vitiligo, nonculture epidermal suspension, melanocyte-keratinocyte transplantation, repigmentation

## Abstract

**Background:**

Vitiligo is a chronic autoimmune disorder characterized by skin depigmentation. Nonculture epidermal suspension has emerged as a promising treatment for stable vitiligo.

**Objective:**

This article evaluates the efficacy and safety of nonculture epidermal suspension in patients with stable vitiligo.

**Materials and Methods:**

Fifty-one patients with stable vitiligo underwent nonculture epidermal suspension treatment between 2016 and 2022. Repigmentation was assessed at 1 month, 3 months, 6 months, and 1 year. Factors affecting outcomes were analyzed.

**Results:**

The mean repigmentation was 68% (95% confidence interval: 62.7–73.9%) with 43.1% of patients achieving excellent repigmentation. Long-term follow-up (> 2 years) in 32 patients showed 78% maintained stable repigmentation. Minor adverse events occurred in 13.7% of patients with no serious complications reported.

**Conclusion:**

Nonculture epidermal suspension is an effective, simple, and safe procedure for stable vitiligo with sustained long-term results.

## Introduction

Vitiligo is a chronic autoimmune disorder characterized by the loss of functional melanocytes, resulting in depigmented patches of skin. It affects approximately 0.5 to 2% of the global population and can significantly impact quality of life causing psychological distress and social stigma. 1 The etiology of vitiligo is multifactorial, involving genetic predisposition, environmental triggers, and autoimmune mechanisms. 2

For patients with vitiligo who have stable disease and where medical management and noninvasive procedures have failed to give results, surgery is useful. These surgical techniques are based on a common principle of pigment transfer and are broadly divided into tissue and cellular grafting. The various tissue grafts include split-thickness skin graft, suction blister graft, punch graft, and hair follicle graft. Tissue graft has its own limitation of covering only a limited surface area per session. Cellular grafts (cultured or noncultured) have the advantage of treating vitiliginous area manifold larger in a single session with small donor skin. However, cultured technique is expensive and takes long time of several weeks for culturing, with safety concerns of additives in the culture medium, 2 these limitations are overcome by noncultured cellular grafting, which gives promising results.

The present study aims to evaluate the long-term efficacy and safety of epidermal cell suspension, identifying factors associated with better outcomes, and to assess the degree of repigmentation achieved through this procedure at various points posttreatment.

## Materials and Methods

### Study Design and Patient Selection

Surgery is indicated for stable vitiligo that does not respond to medical treatment. Patients with stable vitiligo were selected for the study (the Indian Association of Dermatologists, Venereologists, and Leprologists Task Force suggests the absence of the progression of disease for past 1 year and no new lesions for the past 1 year as a definition of stability). The procedure was performed at a single center, over a span of 6 years with an informed consent form, elaborating the procedure. The consent form states the nature of the disease, possible future progression, the vague nature of stability determination, need for medical therapy, and the possibility of repeating the procedure if hypopigmentation or repigmentation does not occur. Donor site was buttocks or waist in all cases and a very thin graft was obtained with Silver's Skin Grafting Knife handle.

### Procedure

**Video 1** Separation of Epidermal Cells by Shaking the Skin Graft.


**Video 2** Separation of Epidermal Cells by Scraping the Skin Graft.


**Video 3** Separation of Dermis from the Epidermis.


**Video 4** Application of Suspension Cell on the Recipient Site.


The procedure was performed under local and/or regional (as per need) anesthesia and a thin split-thickness skin graft was harvested, which was then transferred to 0.25% trypsin-ethylenediaminetetraacetic acid (EDTA) solution in a Petri dish (graft should be immersed in trypsin-EDTA solution with epidermal surface facing upward). This is then incubated at 37°C for 50 to 60 minutes. (By this time the recipient site was dermabraded until pinpoint bleeding was observed.) After incubation of 50 minutes, the Petri dish is taken out from the incubator and trypsin is washed with Ringer lactate solution in a Petri dish and the graft is gently scraped ( Video 1 ) or shaked ( Video 2 ) to separate epidermal cells. Dermis is removed from the epidermis ( Video 3 ). The remaining Ringer lactate solution with epidermal cells (melanocytes and keratinocytes) is then transferred to a sterile test tube, which is then centrifuged at 1,000 revolutions per minute for 10 minutes. Epidermal cells will collect at the bottom of the test tube as pellets. Supernatant was discarded. These pellets were resuspended with hydroxypropyl methylcellulose solution (it is a clear and transparent viscous ophthalmic solution) to form a paste. This paste (cellular suspension) was then applied to the recipient site ( Video 4 ) and secured with a now-adherent dressing (collagen and paraffin gauze). Patients were advised to limit movement of the treated area for 30 to 60 minutes for proper cell fixation and another 2 to 3 days instructed to avoid aggressive movement. Follow-up dressing was done after 7 to 10 days.

### Assessment of Repigmentation

Repigmentation was assessed at 1, 3, 6, and 12 months posttreatment, with long-term follow-up (> 2 years) available for a subset of patients. Standardized photographs were taken at each visit for objective assessment. Two independent dermatologists evaluated the photographs to determine the extent of repigmentation and the progression of disease.

The extent of repigmentation was categorized as:

- Excellent: > 90% repigmentation
- Good (acceptable): 50 to 89% repigmentation
- Fair: 25 to 49% repigmentation
- Poor: < 25% repigmentation

## Results

A total of 51 patients with stable vitiligo were included in this study. The cohort comprised of 28 males (54.9%) and 23 females (45.1%) with a mean age of 35.7 years (range 18–62 years). The mean duration of vitiligo prior to treatment was 8.3 years (range: 2–20 years), and the mean affected body surface area was 12.5% (range: 2–30%) ( Table 1 ). The most frequently treated areas were the face and neck (37% of patients), lower limb (25%), and trunk (18%), with the remaining 20% distributed across the body sites ( Table 2 ). Following nonculture epidermal cell suspension transplantation, 43.1% of patients ( *n* = 22) achieved excellent repigmentation (> 90%) while 29.4% ( *n* = 15) showed good (acceptable) repigmentation (50–89%). Fair repigmentation (25–49%) was observed in 17.6% of patients ( *n* = 9), and 9.8% ( *n* = 5) had poor repigmentation (< 25%). The mean repigmentation percentage across all patients was 68.3% (95% confidence interval: 62.7–73.9%) ( Table 3 ).

**Table 1:** Patient demographics and disease characteristics

Characteristic	Value
Total patients	51
Gender	Males: 28 (54.9%), females: 23 (45.1%)
Age	Mean: 35.7 years, range: 18–62 years
Disease duration	Mean: 8.3 years, range: 2–20 years
Affected BSA	Mean: 12.5%, range: 2–30%

Abbreviation: BSA, body surface area.

**Table 2:** Treatment areas

Area	Percentage of patients
Face and neck	37
Lower limb	25
Trunk	18
Other	20

**Table 3:** Repigmentation outcomes

Repigmentation level	Number of patients	Percentage
Excellent (> 90%)	22	43.1
Good (acceptable) (50–75%)	15	29.4
Fair (25–49%)	9	17.6
Poor (< 25%)	5	9.8

Although literature shows better repigmentation in younger age, but in the present study patients who were above 40 years have shown good repigmentation too, which may be related to the stability of the disease. Minor adverse events were reported in 13.7% of patients ( *n* = 7), including mild erythema in 5 patients (9.8%) and transient hyperpigmentation in 2 patients (3.9%). No serious adverse events were observed during the study period. Long-term follow-up (> 2 years) was available for 32 patients with 78% maintaining stable repigmentation. All patients were also under follow-up with dermatologist. Satisfaction was assessed using a 5-point Likert scale. The mean satisfaction score was 4.2 (standard deviation: 0.8) with 82.4% of patients reporting being satisfied or very satisfied with their treatment outcomes.

## Discussion

One of the key strengths of the present study is the long-term followup data, which addresses a crucial gap in the existing literature with 78% of patients maintaining stable repigmentation at > 2 years posttreatment; the results provide strong evidence for the durability of outcomes achieved through nonculture epidermal cell suspension. This finding is particularly important given that the long-term stability of repigmentation has been a concern with other vitiligo treatments. 3 4 These findings not only corroborate the existing literature on potential of nonculture epidermal cell suspension but also offer long-term stability of repigmentation.

**Fig. 1:**
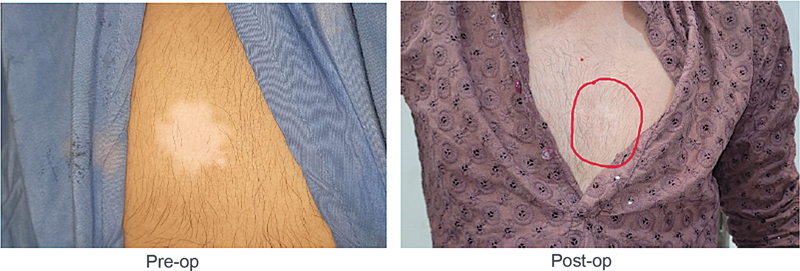
Nine months postop shows almost no scar visible at the presternal region after performing epidermal cell suspension.

Nonculture epidermal cell suspension (autologous melanocyte) is a state of the art procedure that is being practiced in only a few centers and has advantage over culture method, cultured melanocytes treatment that needs 2 to 3 weeks more time and has advantage over other methods like skin grafting, punch grafting, and suction blister grafting as it (nonculture epidermal cell suspension procedure) covers larger area of depigmented patches (1:3 to 1:10) by a small graft. Postprocedure scarring is almost negligible in epidermal cell suspension procedure as compared with ultrathin skin grafting or suction blister grafting. As plastic surgeons, are always afraid of scars over the presternal region. Fig. 1 shows almost no scar at this area after performing epidermal cell suspension procedure, moreover suction blister and ultrathin skin grafting procedure sometimes have hyperpigmented grafted area, which are difficult to treat; with nonculture epidermal cell suspension procedure. In the current study, hyperpigmentation was not observed (except transient hyperpigmentation in few cases) in any of my patients. None of the patients had milia formation, which is almost always seen in skin grafting patients.

**Fig. 2:**
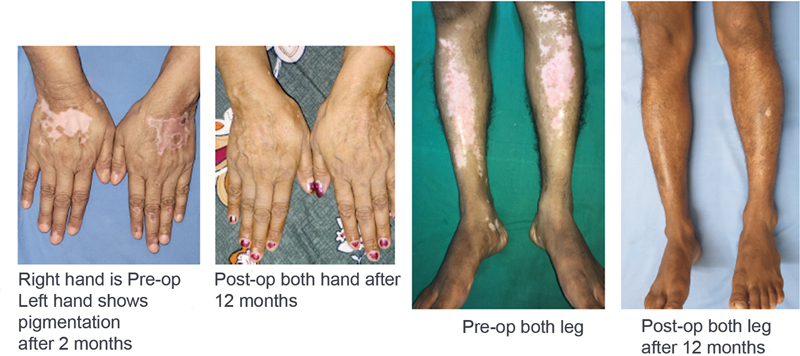
(From left to right) Right hand preop and left hand postop 2 months, both hands 12 months postop, both legs preop, both legs 12 months postop.

Safety profile of nonculture epidermal cell suspension in this study was highly favorable, with only minor adverse events reported in 13.7% of patients and no serious complications. This low rate of adverse events is consistent with other studies on epidermal cell suspension treatment 5 and underscores the safety of the procedure when performed by experienced practitioners. The high satisfaction rate observed in this study suggests that it not only provides clinical improvement but also positively impacts patients' perception of their condition and treatment outcomes. The overall repigmentation outcomes this my study are notably encouraging with 43.1% of patients achieving excellent repigmentation. This success rate is comparable to or slightly higher than those reported in previous studies. 6 For instance, Mulekar 7 reported a success rate of 56% in their series of 142 patients, while Komen et al 8 found 71% of patients achieved > 75% repigmentation in their study of 178 patients. The mean repigmentation rate of 68.3% further supports the effectiveness of this procedure in treating stable vitiligo. 9 Although literature suggests better results in segmental vitiligo, the present study shows good repigmentation in vitiligo vulgaris patients too ( Fig. 2 ). Previous literature mentions the need of laboratory setup and costly equipment and reagents for the procedure to be performed, but in our study an operation theater provided sterile environment for the procedure and the reagents and incubator used are definitively not costly.

Dulbecco's modified Eagle's medium (DMEM) contains a fourfold higher concentration of amino acids and vitamins with additional supplementary components used for cell viability during the procedure while separating epidermal cells in Petri dish. 2 It was used initially in a few cases; however, it was found to be unnecessary because, after centrifugation, the pellets at the bottom were collected and the DMEM medium was discarded. Many physicians used DMEM media for cell suspension to be spread over derma braded area, 2 but my study found hydroxypropyl methylcellulose solution to be far better than this media as it is more viscous than DMEM and we have stopped using DMEM after 6 months of my study.

**Fig. 3:**
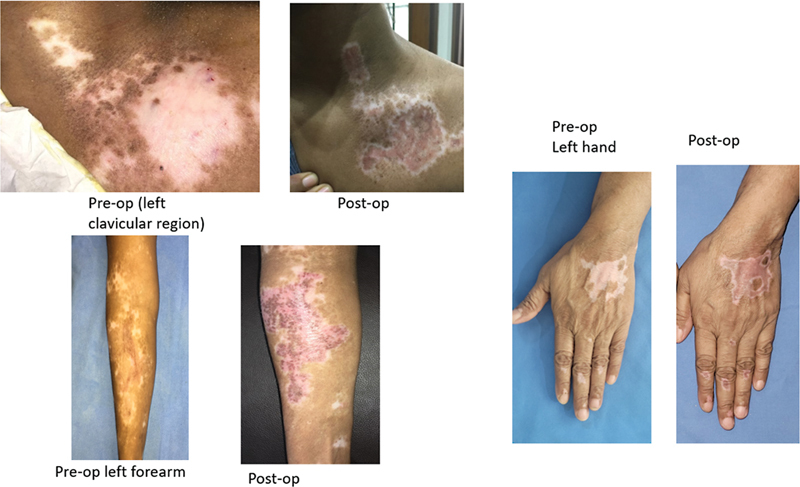
Shows uniform color after 2 months of procedure, appearing hypopigmented in almost all cases with halos over treated area.

**Fig. 4:**
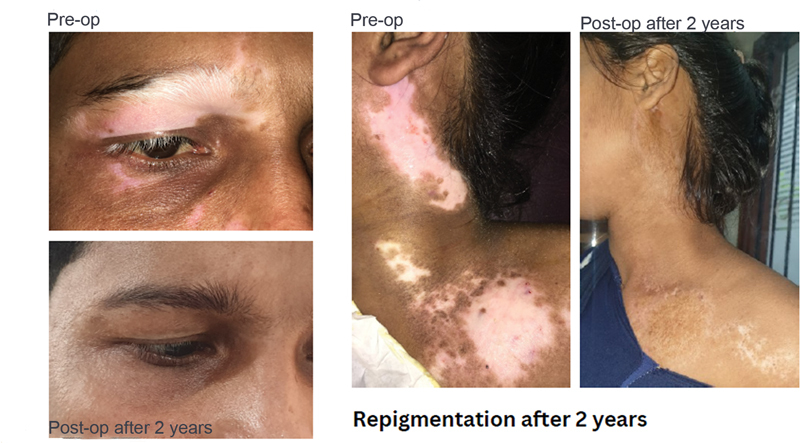
Shows excellent repigmentation of the procedure 2 years postoperatively.

During the first follow-up, after 7 to 10 days, when the dressing was removed, the treated area appeared bright pink in color and repigmentation was seen in few cases as early as 14 days but usually after 3 to 4 weeks. It reached uniform color after 2 months and appears hypopigmented in almost all cases with halos over treated area ( Fig. 3 ). The overall repigmentation outcomes in this study are notably encouraging with 72.5% of patient achieving excellent repigmentation ( Fig. 4 ), which is comparable to the study of Mutalik and Rasal 10 and Donaparthi and Chopra. 11 One patient, aged 10 years, with segmental vitiligo over the trunk and another patient aged 30 years with segmental vitiligo over the forehead did not show any repigmentation of depigmented area, although the procedure was done twice at an interval of 6 month, which were then treated by ultrathin skin grafting. Note that fifteen to twenty percent of the patients showed hypopigmentation and the procedure was repeated in some of these patients who were not satisfied with repigmentation for better aesthetic results.

This study lacks staining of melanocytes with Trypan blue and counting it simultaneously with Neubauer chamber. 1 Good repigmentation after few months of the procedure of the treated vitiliginous area itself shows that viable cells were been transplanted. Also, approximately 1,000 to 1,500 cells/mm ^²^ of the recipient were required 12 ; however, this measurement was not assessed in the present study. Over time, it was understood that for the face, a skin expansion ratio of 1:3 is required, whereas for the trunk and limbs, an expansion ratio of 1:4 or 1:5 is needed; more than this (1:6 to1:10) may result in hypopigmentation. This procedure is performed by a single surgeon at one center, so there are chances of biased results.

Melanocyte density (i.e., number of melanocytes per square cm) is the same in individuals of different races and thus cutaneous pigmentation does not depend on melanocyte numbers but it depends upon melanogenic activity (i.e., the proportion of mature melanosomes) and their transfer (melanosomes are transported along the dendrites of melanocytes) and distribution within keratinocytes. Under normal condition there is roughly one melanocyte for every 36 keratinocyte and each keratinocyte in human skin can receive thousands of melanosomes from melanocytes and regular exposure to sunlight can stimulate melanin production and enhance pigmentation process.

## Conclusion

Nonculture epidermal cell suspension treatment in a stable vitiligo is safe, simple, and has shown better results over the other surgical modalities with an advantage over conventional skin grafting as it requires less donor skin, imparts homogeneous pigmentation over recipient site, less scarring, and reduced chances of hypertrophic scar.
